# A randomized controlled study on medical students learning anatomy through hands‐on ultrasound

**DOI:** 10.1002/ase.70078

**Published:** 2025-07-02

**Authors:** Micah Grubert Van Iderstine, Jacob Jensen, Tomislav Jelic, Terry Y. Li

**Affiliations:** ^1^ Max Rady College of Medicine, University of Manitoba Winnipeg Manitoba Canada; ^2^ Department of Emergency Medicine Max Rady College of Medicine, University of Manitoba Winnipeg Manitoba Canada; ^3^ Department of Human Anatomy and Cell Science Max Rady College of Medicine, University of Manitoba Winnipeg Manitoba Canada

**Keywords:** anatomic imaging, anatomy, anatomy education, radiology, randomized controlled study, ultrasound

## Abstract

Anatomy education in the undergraduate medical curriculum faces many competing interests, including the increasing demand for ultrasound learning. Instead of being treated as a separate subject, ultrasound can offer a unique lens to visualize anatomy without using cadaveric materials. This study used a randomized controlled design to compare learning with hands‐on ultrasound to learning on cadavers. Forty preclinical medical students were randomized to an experimental group or a control group. Both groups attended a session covering structures in the neck, upper limb, and abdomen. The experimental group learned using hands‐on ultrasound imaging, while the control group was taught on a dissected cadaver. Participants completed multiple‐choice tests on anatomical relationships and structure identification at four time points: presession, post‐session, 1‐week follow‐up, and 1‐month follow‐up. There was no statistical difference between test scores of the two groups. The post‐session average score (54% for cadaver group, 57% for ultrasound group) more than doubled the presession average score (20% for cadaver group, 25% for ultrasound group) (*p* < 0.001). One‐week follow‐up and 1‐month follow‐up scores (40%–44%) significantly decreased from immediate post‐session for both groups. Eighteen of the 20 ultrasound‐facilitated participants felt more confident operating an ultrasound device compared to before the session. Three quarters of all participants agreed hands‐on ultrasound should be part of their anatomy education. This study shows hands‐on ultrasound imaging can effectively substitute cadaver‐based learning of certain anatomy topics at the introductory level. Ultrasound integration highlights the clinical relevance of anatomy and provides an innovative tool for anatomical education.

## INTRODUCTION AND BACKGROUND

While ultrasound has become increasingly recognized as an important tool for physicians in many specialties, the incorporation of ultrasound into undergraduate medical education has been slow and variable, without widely adopted strategies.^1^, ^2^, ^3^, ^4^ Some common approaches to ultrasonography education include teaching students at the clerkship level (in their third and fourth year of undergraduate medical education) during their clinical rotations, providing a small number of focused tutorials throughout undergraduate medical training, and providing didactic lectures that discuss ultrasound physics and common pathological images.^5^ Unfortunately, numerous barriers have prevented many medical schools from developing complete ultrasound curricula to date.^6^

The major barriers to implementing ultrasound education in medical schools include a lack of equipment and time in current curricula.^2^, ^4^ Advances in technology have made ultrasound available in a pocket‐sized form factor, readily connected to smart phones or tablets. Handheld ultrasound (HHUS) devices are the key to overcoming the equipment barrier, as they have become more affordable and capable, and can be easily adapted to different learning environments.^7^, ^8^

Hands‐on ultrasound at an undergraduate medical education level may be able to serve a dual purpose as an effective anatomy teaching tool, while providing early exposure to an invaluable clinical skill. HHUS devices are known to help clinicians answer focused clinical questions, guide treatment decisions at the bedside, and are an effective adjunct to the clinical exam.^9^, ^10^ It has been suggested that early exposure to hands‐on ultrasound in the undergraduate medical education curriculum may help to improve student clinical decision‐making skills and promote the development of strong ultrasonography skills.^11^

Previous studies have shown that hands‐on ultrasound experience can enhance anatomy knowledge among undergraduate medical students.^12^, ^13^, ^14^ Comparative studies evaluating the effectiveness of teaching cardiac and joint anatomy with ultrasound suggest that it may be possible to replace parts of the traditionally cadaveric‐based curriculum with ultrasound imaging.^15^, ^16^ Notably, student sentiment regarding the use of ultrasound to learn anatomy has been consistently positive.^12^, ^14^, ^15^, ^17^

While several studies further highlight the benefits of ultrasound curricula, they are limited in their coverage of anatomic structures, and often do not employ a randomized controlled design.^12^, ^13^, ^14^, ^15^, ^16^, ^18^ To date, ultrasound teaching has been largely delivered as an addition to existing anatomical curricula, through supplemental didactic lectures and hands‐on tutorials, rather than integrated into the curriculum.^12^, ^17^, ^18^, ^19^, ^20^ This study will propose that instead of adding it to an already busy curriculum, ultrasound can potentially substitute some time and attention dedicated to traditional anatomy learning centered around cadavers.

When considering a fit for increased ultrasound exposure in curricula, it is important to note anatomy education has long focused on showing the human body through different lenses, and the use of medical imaging in teaching anatomy has become a common practice.^20^, ^21^, ^22^ Proposed ways to increase ultrasound exposure in medical school curricula include using ultrasound to identify normal structures in the first year anatomy courses, with more detail and abnormal findings being added in each subsequent year.^5^ In addition to learning relevant clinical anatomy, students who are exposed to hands‐on ultrasound in interactive sessions can learn the skills of how to hold the ultrasound transducer, generate optimal images, and interpret those images.^23^

The purpose of this study was to determine if hands‐on ultrasound can be used in the classroom as an effective tool for teaching anatomy and the basics of point‐of‐care ultrasonography to preclinical medical students. The objectives of the study were (1) to develop hands‐on ultrasound and cadaver‐based learning sessions that cover structures in the head and neck, the upper limbs, and the abdomen, selected from an established curriculum^24^; (2) to recruit preclinical medical students and randomly assign them to one of the two learning sessions; (3) to design tests that evaluate anatomical knowledge, which were administered before and after the session, and compare test scores between the hands‐on ultrasound learning session and the cadaver‐based learning session; and (4) to obtain students' perception of the learning sessions and attitude toward learning anatomy with ultrasound.

This randomized controlled design allowed us to determine the efficacy of teaching and learning anatomy with hands‐on ultrasound in comparison with the traditional cadaver‐based anatomical education. We also assessed student perceptions of the learning modules, preferences in pedagogical approach, and their opinions on which anatomical regions are better suited to ultrasound‐based learning.

## MATERIALS AND METHODS

### Study design

This was a randomized controlled trial comparing ultrasound‐based anatomy teaching efficacy to traditional cadaveric teaching methods in medical students. The study was designed and reported in accordance with the CONSORT 2010 statement.^25^ Participants were equally allocated into two parallel interventions that took place during a 2‐year recruitment period. No changes to methods were made throughout the duration of the trial.

### Participants

The research protocol was approved by the Human Research Ethics Board at the University of Manitoba Bannatyne Campus (HS25661). The inclusion criteria for the study were set to include students enrolled in the first 12 months of preclinical training at the University of Manitoba Max Rady College of Medicine. An email invitation including the consent form was sent to students in graduating classes of 2025, 2026, and 2027. These students were at various stages of acquiring knowledge in normal anatomy, physiology, and basic clinical skills. Ultrasound had not been formally introduced in the curriculum at the time of recruitment. Students with prior knowledge in ultrasound were excluded. Participants were self‐selected and took part in the study voluntarily outside of curricular time. They were guaranteed that their performance during the study would not affect their academic standing.

### Sample size

An a priori sample size calculation determined that 16 participants per interventional group would be required to achieve an alpha of 0.05 and a statistical power (1‐beta) of 0.80, to detect a mean difference in knowledge test scores of two or greater.

### Randomization

Participants from each graduating class were randomized as a block to ensure 1:1 allocation in each class. Within each block, participants were randomly assigned to either the control or experimental group by a computer‐generated sequence. The enrollment and randomization were conducted by a researcher who was not involved in teaching. The allocation was concealed from the teaching researchers and participants until the day of the intervention.

### Interventions

Each participant in the experimental group attended a single hands‐on ultrasound learning session, and each participant in the control group attended a single cadaver‐based learning session. Multiple learning sessions were held at different times, so that each session had no more than eight students to simulate a typical small‐group anatomy laboratory session. The content of the learning sessions was supplemental to the pre‐clerkship curriculum.

#### Hands‐on ultrasound learning session

Each session consisted of three anatomical regions. The head and neck region included eye, parotid gland, submandibular gland, sternocleidomastoid muscle, carotid arteries, internal jugular vein, thyroid gland, trachea, and interscalene brachial plexus. The upper limb region included rotator cuff muscles, glenohumeral joint, median nerve, brachial artery, radial nerve and artery, ulnar nerve and artery, and carpal tunnel. The abdominal region included liver, gallbladder, spleen, kidneys, abdominal aorta and its major branches, inferior vena cava, and portal vein. Participants in the ultrasound learning group identified these structures using a set of Clarius HD HHUS devices (Vancouver, BC, Canada) on a volunteer model (previously consented and screened for normal anatomy). Every participant scanned the model for 5–10 min in each region while the rest of the small group observed. An anatomist proficient in ultrasound imaging demonstrated scanning techniques and facilitated participant identification of structures and understanding of anatomical relationships. Each session took approximately two hours to complete.

#### Cadaver‐based learning session

The cadaver‐based session was structured identically to the ultrasound session. Participants in the control group explored the same structures as listed above on fully dissected whole‐body human cadavers. These cadavers were acquired through the Body Donation program and embalmed with 10% formalin. Cross‐sectional images of cadavers were provided to supplement learning. The same anatomist who led the ultrasound learning session demonstrated cadaveric structures and their anatomical relationships. The content of both ultrasound and cadaver‐based sessions was developed in conjunction by an anatomist and an Emergency Medicine physician. The complete list of structures was provided to participants from both groups during the session. No additional learning material was supplied before or after the session.

### Outcome evaluation

The primary outcome was anatomical knowledge assessed by multiple‐choice format tests administered immediately after the learning session, compared to anatomical knowledge at baseline before the learning session. Knowledge retention was also examined by tests administered at 1‐week follow‐up and at 1‐month follow‐up. The first 12 questions of each test examined the understanding of anatomical relationships, the following six questions asked participants to identify structures in ultrasound images, and the final six questions required identification of structures on photos of dissected cadavers. Each question consisted of a single correct response option, four distractors, and an “I don't know” option to prevent participants from guessing. While the questions used in each knowledge test were not identical, they were checked for difficulty consistency, face validity, and content validity by an independent anatomist. An example knowledge test is included in the supplementary material.

The secondary outcome was participants' perception of their confidence in anatomical knowledge and the effectiveness of the learning sessions. This was evaluated immediately after the learning session using a Likert scale questionnaire and optional open‐ended comments (included in the supplementary material). All tests and questionnaires were administered via Google Forms.

### Data analysis

The scores for the pretest, posttest, 1‐week test, and 1‐month test were calculated for each participant and compared between both learning groups and across time points using a repeated measures ANOVA test. Data heteroscedasticity was confirmed by graphical methods, and a Shapiro–Wilk test was performed to confirm the data were normally distributed, confirming the data met the assumptions for an ANOVA test. Post hoc testing consisted of paired Student's *t*‐tests between the test scores at various time points, with a Bonferroni correction applied to the *p*‐values. This analysis was repeated for each of the question type and anatomical region subgroups that we identified for analysis.

All statistical analysis and data visualization were performed using R version 4.2.2. The central tendency used for parametric continuous data is the mean, and the measurement of variation shown in brackets is the standard error. Demographic information was compared by Student's *t*‐test or chi‐square test as required. Responses to the Likert scale questionnaire were tabulated and visualized. A statistical comparison of Likert scale responses between groups was conducted using the Mann–Whitney *U* test.

## RESULTS

Forty preclinical medical students were recruited and randomized in three blocks that took place in fall 2022, spring 2023, and fall 2023. The allocation of participants to the cadaver‐based learning group and the hands‐on ultrasound learning group was 20 and 20. Within each block, the full intervention was completed over a 1‐month period, from the pretest and learning session to the 1‐month follow‐up test. There was no loss or exclusion of participants after randomization. Recruitment was terminated when the required sample size of 16 per group was exceeded.

The demographic data of participants is summarized in Table 1. There was no statistically significant difference in age, gender, educational background, or level of training between the two interventional groups.

**TABLE 1:** Demographic data at the time of randomization.

	Cadaver‐based learning group (*n* = 20)	Hands‐on ultrasound learning group (*n* = 20)	*p*‐Value
Mean age	25 years	25 years	0.968 (*t* = 0.04)
Female gender	11	9	0.527 (*X* ^2^ = 0.4)
Mean months of medical training	4.5 months	4.8 months	0.823 (*t* = −0.22)

**FIGURE 1:**
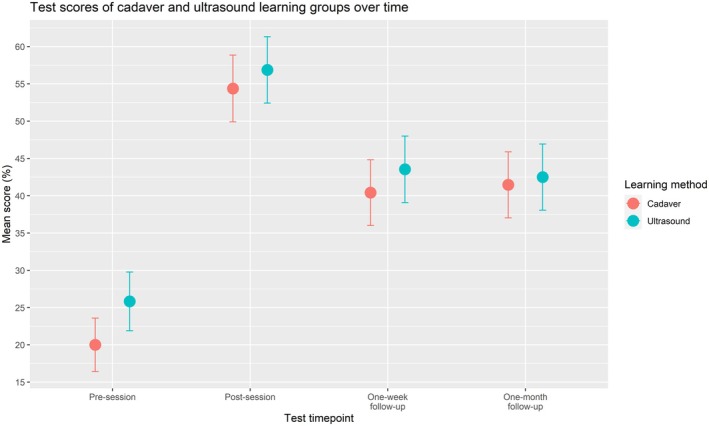
Mean knowledge test scores at each time point throughout the study are shown in red for the cadaver‐based learning group, and blue for the ultrasound‐based learning group. Whiskers represent the 95% confidence intervals for each of the scores.

The mean baseline knowledge test scores were 25.8% (±2.0%) for the ultrasound group, and 20.0% (±1.8%) for the cadaver group. Immediately following the learning session, the knowledge test scores increased to 54.4% (±2.3%) and 56.9% (±2.3%) for the ultrasound and cadaver groups, respectively. One week following the learning sessions, the knowledge test score for the ultrasound group decreased to 43.5% (±2.3) while the test score for the cadaver‐based group decreased to 40.4% (±2.2%). On the final knowledge test which took place one month after the learning session, the ultrasound group scored 42.5% (±2.3%) and the cadaver group 41.5% (±2.2%). These results are visualized in Figure 1.

A repeated measures ANOVA test analyzing the effect of learning modules and time points on test score showed there was no significant difference in knowledge test scores between ultrasound and cadaver‐based learning groups at any time point throughout the study (*p* = 0.558), although it did show a significant difference in test scores at different time points (*p* ≪ 0.005). A post hoc pairwise *t*‐test with a Bonferroni correction applied revealed that there was a significant difference between the presession test score and post‐session test score (*p* < 0.005); the 1‐week and 1‐month follow‐up test scores were both significantly higher than presession test scores and significantly lower than post‐session test scores (*p* < 0.005). There was no significant difference between the 1‐week and 1‐month follow‐up scores (*p* = 1.00).

**FIGURE 2:**
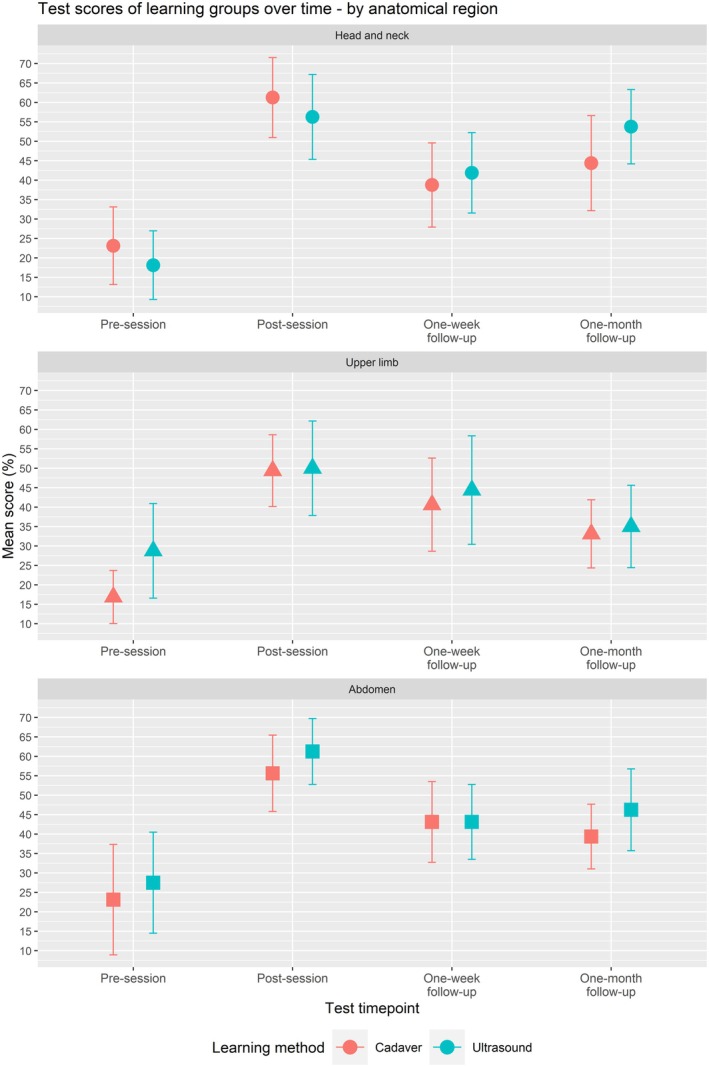
Mean knowledge test scores for specific anatomical regions (head and neck, upper limb, and abdomen) at each time point throughout the study are shown in red for the cadaver‐based learning group, and blue for the ultrasound‐based learning group. Whiskers represent the 95% confidence intervals for each of the scores.

**FIGURE 3:**
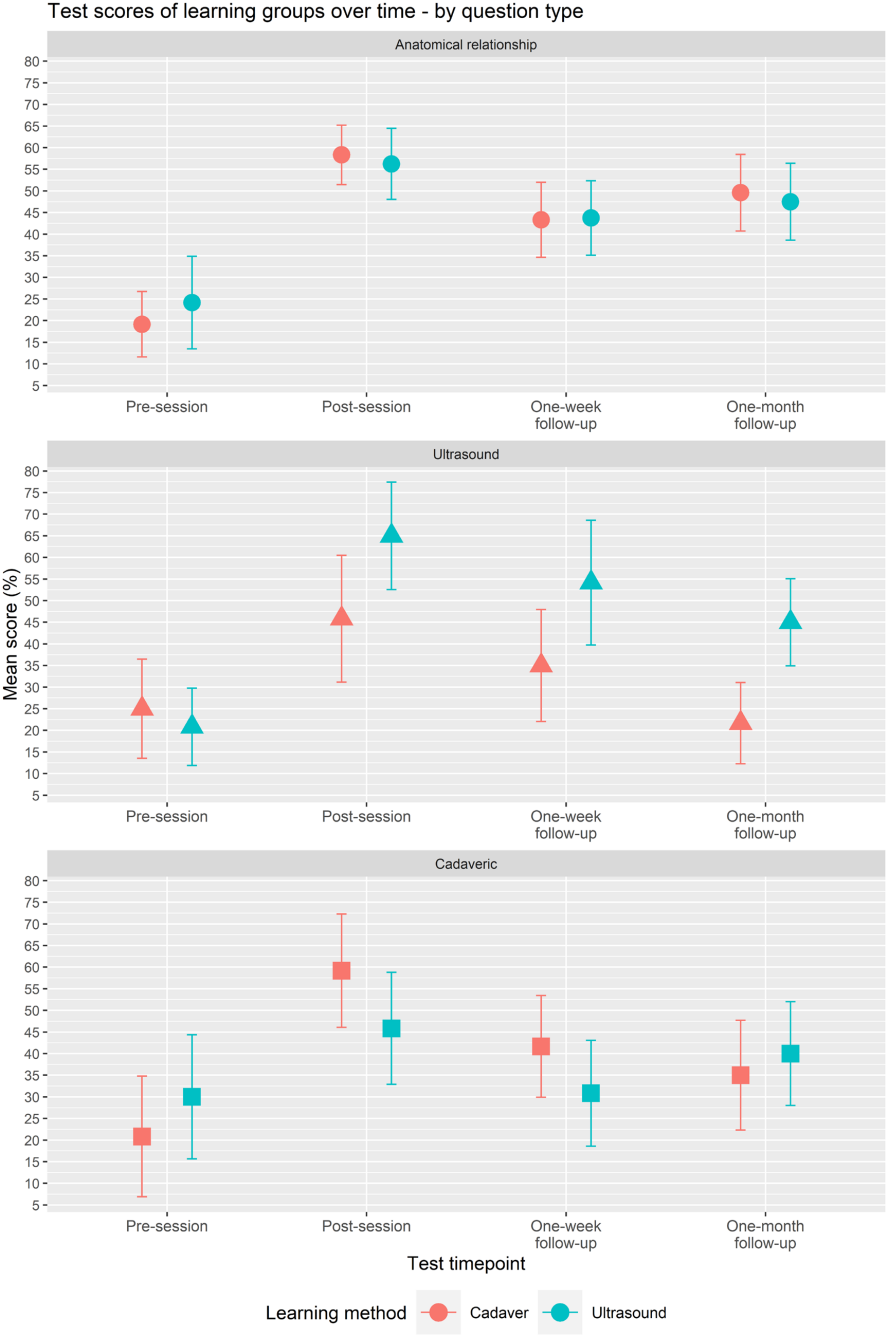
Mean knowledge test scores for specific question types (anatomical relationship, ultrasound, and cadaveric) at each time point throughout the study are shown in red for the cadaver‐based learning group, and blue for the ultrasound‐based learning group. Whiskers represent the 95% confidence intervals for each of the scores.

Sub‐analyses showed that test scores were significantly different at different time points for all three anatomical regions and all three question types. The trends of test score over time were similar to the overall analysis and are visualized in Figure 2 for anatomical region and Figure 3 for question type. There was no significant difference in test scores between learning modules across anatomical regions and question types, except for questions on identifying structures in ultrasound images (*p* = 0.034). For these questions, the ultrasound‐based learning group scored significantly higher than the cadaver‐based learning group at post‐session, 1‐week follow‐up, and 1‐month follow‐up time points. The repeated measures ANOVA results are summarized in Table 3 for anatomical region and Table 4 for question type.

The test scores and mean differences between them at each time point are shown in Table 2. The test scores and 95% confidence intervals over time are broken down into the anatomical region they are relevant to and can be seen in Figure 2. Question by question performance is visualized in Appendix S1.

**TABLE 2:** Summary of mean student test scores and unknown question answers at each time point throughout the study.

Learning group	Knowledge test time point	% of “I do not know” responses	Mean score (%)	Standard error (%)	Upper 95% confidence interval (%)	Lower 95% confidence interval (%)
Ultrasound	Pre/Baseline	58.3	25.8	2.0	29.8	21.9
Cadaver	Pre/Baseline	64.2	20.0	1.8	23.6	16.4
Difference	Pre/Baseline	−5.9	5.8			
Ultrasound	Post	18.8	56.9	2.3	61.3	52.4
Cadaver	Post	18.8	54.4	2.3	58.9	49.9
Difference	Post	0	2.5			
Ultrasound	1 week Post	27.1	43.5	2.2	48.0	39.1
Cadaver	1 week Post	32.1	40.4	2.2	44.8	36.0
Difference	1 week Post	−5.0	3.1			
Ultrasound	1 month Post	28.1	42.5	2.3	46.9	38.1
Cadaver	1 month Post	37.5	41.5	2.3	45.9	37.0
Difference	1 month Post	−9.4	1.0			

*Note*: “Difference” shows the difference between the ultrasound and cadaver group scores.

The repeated measures ANOVA results analyzing the effects of learning modules and time points on scores for types of questions and on scores for questions pertaining to different anatomical regions are displayed in Tables 3 and 4, respectively. A Shapiro–Wilk test showed the data were likely to be from a normal distribution (*p* = 0.10). In the question type sub‐analysis, the test scores were significantly different at different time points in all question types (*p* < 0.005). Ultrasound‐based questions were the only type of question where the learning group was a significant factor (*p* = 0.034). As seen in Table 4, there were no significant differences between the scores by those participating in the different learning modules for any of the anatomical regions tested. However, the scores did vary over the different time points in each of the three anatomical regions tested.

**TABLE 3:** Results of repeated measures ANOVA tests comparing knowledge test scores by learning module over time for each anatomical region covered in the modules.

Anatomical region				
		Degrees of freedom	*F*‐score	*p*‐Value
Head and neck	Interaction (Learning module:Time point)	3, 114	1.828	0.157
	Main effect (Learning module)	1, 38	0.014	0.905
	Main effect (Time point)	3, 114	39.534	6.03E−15*
Upper limb	Interaction (Learning module:Time point)	3, 114	1.101	0.352
	Main effect (Learning module)	1, 38	0.597	0.444
	Main effect (Time point)	3, 114	22.977	1.05E−11*
Abdomen	Interaction (Learning module:Time point)	3, 114	0.36	0.782
	Main effect (Learning module)	1, 38	0.57	0.455
	Main effect (Time point)	3, 114	29.511	3.40E−14*

* Statistically significant.

**TABLE 4:** Results of repeated measures ANOVA tests comparing knowledge test scores by learning module over time subdivided by question type.

Question type				
		Degrees of freedom	*F*‐score	*p*‐Value
Anatomical relationship questions	Interaction (Learning module:Time point)	3, 114	0.74	0.946
	Main effect (Learning module)	1, 38	0.005	0.53
	Main effect (Time point)	3, 114	61.055	1.30E−23*
Cadaveric questions	Interaction (Learning Module:Time point)	3, 114	3.451	0.019*
	Main effect (Learning module)	1, 38	0.139	0.711
	Main effect (Time point)	3, 114	13.539	1.29E−07*
Ultrasound questions	Interaction (Learning module:Time point)	3, 114	6.056	7.30E−04*
	Main effect (Learning module)	1, 38	4.83	0.034*
	Main effect (Time point)	3, 114	30.485	1.52E−14*

* Statistically significant.

**FIGURE 4:**
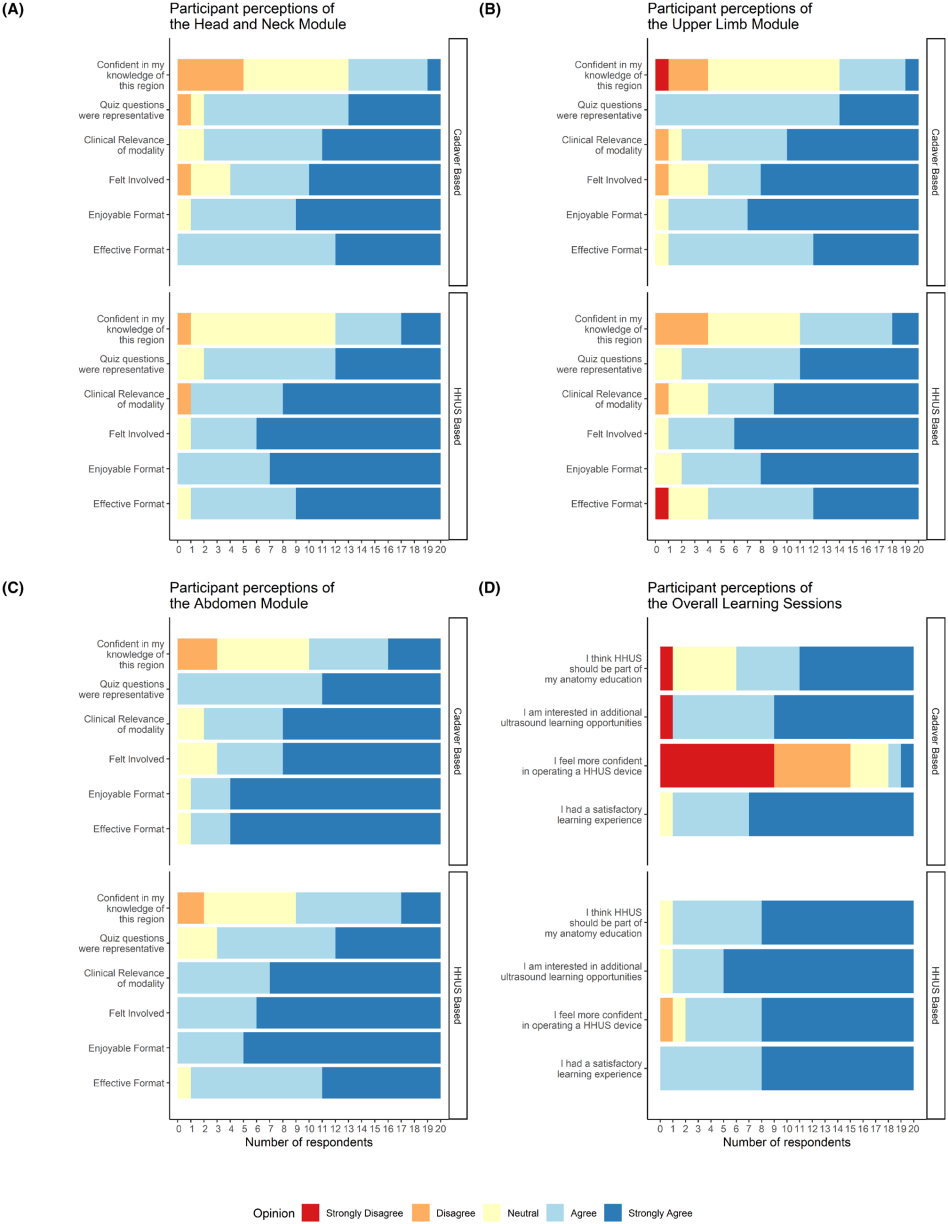
Participant responses to Likert scale questions regarding the head and neck region (A), upper limb region (B), abdomen region (C), and the overall learning sessions (D) separated by learning methods.

The post‐session Likert scale questionnaire responses are visualized in Figure 4. In the abdominal region, there was a significant difference in opinion regarding the efficacy of the teaching format used in each group (*U* = 56.5, *p* = 0.036, Appendix S2). There was also a significant difference in confidence operating an ultrasound device between the two groups (*U* = −183.5, *p* < 0.001, Appendix S2). The remainder of the comparisons of Likert responses are shown in Appendix S2.

Since the open‐ended portion of the post‐session questionnaire was optional, only 13 out of 40 participants provided comments (eight from ultrasound group and five from cadaver group). Most of them expressed that the learning session was informative, interactive, and engaging. Some notable or critical comments were:

I believe that POCUS can empower students to have a more thorough understanding of their anatomy as it allows for a unique dynamic perspective that I believe could not be achieved with cadaveric‐style learning alone.—Study ID #15 (ultrasound group)

I found that it was more difficult for me to learn the muscles with ultrasound as compared to cadaver, everything else was very useful to learn with US and clinically applicable.—Study ID #11 (ultrasound group)

US of shoulder region was confusing due to the large number of axes and views used.—Study ID #22 (ultrasound group)

This method of visualizing internal structures is great, especially for vessels because a learner gets to see things in real time. Also, learners get to see internal structures in relation to external positioning.—Study ID #31 (ultrasound group)

I didn't get an opportunity to try the ultrasound, but I would've very much enjoyed to have tried. I think labs should be a combo of ultrasound and cadaver learning and we should have more opportunities in Med 2 for lab exposures in anatomy.—Study ID #2 (cadaver group)

I would do differently next time (for myself) to run through every major part again at the very end to see how they all work together to form that area and not look at them in isolation. Would've liked to try ultrasound too!—Study ID #30 (cadaver group)

## DISCUSSION

This study used a randomized controlled design to demonstrate that preclinical medical students learned anatomy through hands‐on ultrasound imaging as effectively as on prosected cadavers. The study design and major findings closely mirrored those of a previous study conducted by Griksaitis et al., who focused on cardiac anatomy and echocardiography; however, our study expanded the scope of anatomic structures to three regions across the body.^16^ In addition to assessing anatomical knowledge immediately after the learning session, this study included two follow‐up time points to evaluate knowledge retention.

The efficacy of the ultrasound‐mediated anatomy learning is further demonstrated by sub‐analysis by question type. Immediately after learning sessions, participants in the ultrasound learning group scored as well as those in the cadaver learning group on anatomical relationship questions, significantly better on ultrasound image identification questions, and worse on cadaveric image identification questions by a statistically insignificant amount. The ultrasound learning group's improvement in the ability to recognize structures in cadaveric photos represents a level of knowledge translation we did not expect.

Compared to previous research, a unique feature of the current study is that participants in the ultrasound learning group performed ultrasound scanning themselves. In order to identify the target structures, participants had to identify the surface anatomy, practice manipulating the ultrasound transducer, and convert two‐dimensional imaging to three‐dimensional anatomical understanding. Even though these students were completely novice to ultrasound imaging, they were able to handle the psychomotor strain of acquiring a new skill and the cognitive strain of grasping anatomy simultaneously. It is possible that instead of competing for mental capacity, these two processes of learning are complementary to each other. This is becoming a more common opinion among medical educators, and we expect this will be widely accepted in the future.^12^, ^14^, ^26^, ^27^ Our results agree with the other studies that have suggested ultrasound can be efficacious in teaching anatomy.^27^, ^28^, ^29^, ^30^ This suggests that ultrasound can effectively substitute cadaver‐based anatomy learning in certain situations, and at a minimum can be used as an adjunct.

Learning retention was similar across both learning modules, with there being a significant drop‐off in knowledge test performance in both modules as time passed after the session, but no significant difference between the two modules at any time point. One previous study has shown that ultrasound electives in medical school may improve retention of basic anatomical knowledge, but no studies have been able to assess the effect of using ultrasound as an adjunct to traditional anatomical teaching modalities.^13^ Our study is the first to determine there is no significant difference in retention of anatomical knowledge between ultrasound‐based and traditional cadaver‐based learning methods, although further studies with larger sample sizes may be needed to confirm this is the case.

A surprising finding was that students learning from the cadaveric module were able to apply their knowledge to ultrasound‐based questions with some success immediately after the learning session. One month later, however, there was a significant drop in performance on these questions (Figure 3, Table 4). Those participating in the ultrasound modules continued to retain their ultrasound‐specific knowledge 1 month after their learning session. Interestingly, the students from the ultrasound module showed a significant knowledge gain with some retention on cadaveric questions in addition to the increase we expected to see on the ultrasound‐based questions. These findings suggest that learning anatomy via ultrasound may allow for better application of knowledge across a variety of media, and thus that they gained a more comprehensive understanding of the anatomy. However, while the knowledge gained by the students in the ultrasound module may have been slightly more versatile, students who learned on cadavers were able to identify structures in ultrasound images. This demonstrates that a solid basis in anatomical knowledge can assist in ultrasound interpretation, even without formal ultrasound instruction.

While previous research focused on a specific organ of the body such as the heart^16^ or joints,^15^ our study selected three anatomical regions, head and neck, upper limb, and abdomen, due to their applicability to primary care, competency in which is the principal goal for undergraduate medical education.^15^, ^16^ These three regions also consist of a varied assortment of organs and structures, ranging from bone to soft tissue to blood vessel, which elucidated strengths and weaknesses of ultrasound‐mediated anatomy learning. Student performance was similar across all three of these regions in both learning groups (Table 3), and no questions were answered incorrectly by all members of a learning group (Appendix S1), meaning that question design was well balanced.

Within each anatomical region, there was no significant difference in test score between learning with ultrasound and on cadaver, and thus, we can conclude that both modalities are comparable in terms of utility for teaching a wide variety of anatomical structures (Figure 3). However, some structures were better visualized by ultrasound imaging than on a cadaver. For example, in anatomy courses at our institution, structures in the eye are typically taught with enlarged plastic models since cadaveric eyes are small and difficult to properly preserve. Ultrasound can “magnify” eye structures and depict dynamic movements that aid learning. Previous reports have also suggested the head and neck region is an effective subject for medical imaging‐based anatomy learning; however, the use of ultrasound has not been reported due to its relatively limited field of view.^31^ Additionally, some comments from students suggested they enjoyed the living anatomy aspect of ultrasound, which can capture phenomena, such as muscle contraction and arterial pulsation that a cadaver cannot simulate.

Visualizing anatomy with ultrasound is not without its limitations. Long structures such as muscles and blood vessels that can be easily followed from one end to the other on a dissected cadaver only show as cross‐sectional slices in ultrasound images. Following long structures or maneuvering around large structures with the ultrasound transducer can be challenging for novice learners. This might explain the Likert scale response where the cadaver‐based session was perceived to be more effective than the ultrasound session for the abdomen region. Four out of 20 participants reported that ultrasound was not an effective format for learning upper limb anatomy. It should be noted, however, that most participants (80%) in both groups agreed or strongly agreed with the effectiveness of the learning sessions for all three regions (Figure 4A–C).

In the Likert scale responses relating to the overall session, participants in the ultrasound group reported significantly higher confidence in handling a HHUS device compared to the cadaver group. Confidence is crucial for the continuous practice and longitudinal development of clinical skills. Instilling confidence in ultrasound scanning as early as the first few months of medical school would be instrumental to becoming proficient in such a ubiquitous tool in primary care and many specialties. Furthermore, students in both groups indicated to a great degree that they felt hands‐on ultrasound should be a part of their anatomy education, and there was no significant difference between the groups (*p* = 0.149, Appendix S2). This aligns with a previous study that shows students place a high value on learning this clinical skill, as they feel it presents an opportunity for skill development that will help them grow in their future practice.^32^ The positive sentiments of students regarding ultrasound in both learning groups may be influenced by the relative novelty of the opportunity to learn sonography. Additionally, students who volunteered to participate in the study may have had a preexisting interest in sonography or radiology.

Another consideration regarding ultrasound‐based learning is its time‐consuming nature. Based on our anecdotal experience, students generally took longer to identify structures with ultrasound scanning than on a cadaver. In the time‐scarce medical school curriculum, it is possible that smaller group sizes and a greater ratio of ultrasound devices to students may be needed to cover the same amount of content in the same amount of time as it takes for cadaver‐based learning groups.

In medical schools, preceptor time is often a scarce resource, so it is a valid concern that incorporating ultrasound into the anatomy curriculum may require significant assistance from trained clinicians which may make teaching ultrasound untenable. In this study, the ultrasound learning sessions were led by an anatomist who was trained by clinicians and experienced in ultrasound teaching. A study by Jurjus et al. showed there was no significant difference in student performance in anatomy questions whether taught by a clinician or anatomist,^30^ which is confirmed by our anecdotal experience. Anatomists who are well versed in ultrasound could alleviate the shortage in clinical preceptors and supplement with an anatomical perspective in ultrasound.

### Limitations

Some important limitations of this study include the sample size, the self‐selection of students, and length of learning period. It is possible that discrepancies in learning may emerge when larger sample sizes are used, although an ad hoc sample size analysis showed a test group sample of 16 participants would be sufficiently powerful to show a statistical difference in test performance between the groups at any time point. Self‐selection of students may introduce a source of bias, as an increased interest in ultrasound relative to the average student may falsely increase their perceived usefulness of the modality for teaching. Another limitation in this study is the lack of a thematic analysis conducted on the open‐ended responses, which was due to the sparsity of these data. In future, a larger sample size, or mandatory response fields may provide sufficient information to conduct a more thorough qualitative analysis.

Additionally, while this study has proven that ultrasound is an effective modality for teaching anatomy in relatively granular detail, it is possible that over the length of a full anatomy course that differences in student understanding and performance may emerge as the level of detail covered increases. While we believe that the single learning session design was effective for answering our experimental question, we acknowledge it is not representative of the real‐world teaching approach that encourages repetition. In future studies, we hope to take a longitudinal approach that can better evaluate student learning and retention over a longer period where repetition can be incorporated into the curriculum.

## CONCLUSIONS

Our results show that learning anatomy with hands‐on ultrasound imaging is as effective as traditional cadaver‐based learning for a variety of structures in head and neck, upper limbs, and the abdomen. Ultrasound‐mediated learning can have more robust knowledge retention. This study demonstrates the feasibility of supplementing introductory anatomy with hands‐on ultrasound imaging, allowing preclinical medical students to explore the living anatomy without cutting the body, while practicing an important clinical skill. Logistical and instructional lessons learned through the study will inform the incorporation of ultrasound teaching in our undergraduate medical curriculum. Future research will investigate multiple sessions, interaction with clinical subjects, and longitudinal effects.

## AUTHOR CONTRIBUTIONS

**Micah Grubert Van Iderstine:** Conceptualization; data curation; formal analysis; methodology; visualization; writing – original draft. **Jacob Jensen:** Conceptualization; data curation; investigation; methodology; project administration. **Tomislav Jelic:** Conceptualization; data curation; investigation; methodology. **Terry Y. Li:** Conceptualization; data curation; investigation; project administration; writing – original draft; writing – review and editing.

## FUNDING INFORMATION

The study was supported by the Max Rady College of Medicine, Dean's Fund and Centre for Teaching and Learning, University of Manitoba.

## CONFLICT OF INTEREST STATEMENT

Nothing to disclose.

## ETHICS APPROVAL STATEMENT

The research protocol was approved by the Human Research Ethics Board at University of Manitoba Bannatyne Campus (HS25661).

## Supporting information


Appendix S1.

Appendix S2.

## Data Availability

The complete dataset is available upon request to the first author (Micah Grubert Van Iderstine, grubertm@myumanitoba.ca).

## References

[1] Bahner DP , Goldman E , Way D , Royall NA , Liu YT . The state of ultrasound education in U.S. medical schools: results of a national survey. Acad Med. 2014;89(12):1681–1686. 10.1097/ACM.0000000000000414 25099238

[2] Steinmetz P , Dobrescu O , Oleskevich S , Lewis J . Bedside ultrasound education in Canadian medical schools: a national survey. Can Med Educ J. 2016;7(1):e78–e86. Available from: http://www.cmej.ca 27103956 PMC4830376

[3] Tarique U , Tang B , Singh M , Kulasegaram KM , Ailon J . Ultrasound curricula in undergraduate medical education: a scoping review. J Ultrasound Med. 2018;37:69–82. 10.1002/jum.14333 28748549

[4] Russell FM , Zakeri B , Herbert A , Ferre RM , Leiser A , Wallach PM . The state of point‐of‐care ultrasound training in undergraduate medical education: findings from a National Survey. Acad Med. 2022;97(5):723–727. 10.1097/ACM.0000000000004512 34789665

[5] Russell FM , Herbert A , Ferre RM , Zakeri B , Echeverria V , Peterson D , et al. Development and implementation of a point of care ultrasound curriculum at a multi‐site institution. Ultrasound J. 2021;13(1):9. 10.1186/s13089-021-00214-w 33615390 PMC7897586

[6] Glass C , Sarwal A , Zavitz J , Nitsche J , Joyner J , Johnson LL , et al. Scoping review of implementing a longitudinal curriculum in undergraduate medical education: the wake forest experience. Ultrasound J. 2021;13(1):23. 10.1186/s13089-021-00206-w 33871741 PMC8055803

[7] Wilkinson JN , Saxhaug LM . Handheld ultrasound in training: the future is getting smaller! J Intensive Care Soc. 2021;22(3):220–229. 10.1177/1751143720914216 34422105 PMC8373282

[8] Baribeau Y , Sharkey A , Chaudhary O , Krumm S , Fatima H , Mahmood F , et al. Handheld point‐of‐care ultrasound probes: the new generation of POCUS. J Cardiothorac Vasc Anesth. 2020;34:3139–3145. 10.1053/j.jvca.2020.07.004 32736998 PMC7340048

[9] Kobal SL , Trento L , Baharami S , Tolstrup K , Naqvi TZ , Cercek B , et al. Comparison of effectiveness of hand‐carried ultrasound to bedside cardiovascular physical examination. Am J Cardiol. 2005;96:1002–1006. 10.1016/j.amjcard.2005.05.060 16188532

[10] Mouratev G , Howe D , Hoppmann R , Poston MB , Reid R , Varnadoe J , et al. Teaching medical students ultrasound to measure liver size: comparison with experienced clinicians using physical examination alone. Teach Learn Med. 2013;25(1):84–88. 10.1080/10401334.2012.741535 23330900

[11] Zoll K , Kondrashov P , Pazdernik V , Beatty D , Arseneaux M , Atieh T , et al. Medical student perception of the impact of early ultrasonography education on experiences during clinical rotations. Med Sci Educ. 2017;27(2):273–280. 10.1007/s40670-017-0394-4

[12] Dreher SM , Dephilip R , Bahner D . Ultrasound exposure during gross anatomy. J Emerg Med. 2014;46(2):231–240. 10.1016/j.jemermed.2013.08.028 24113480

[13] Kondrashov P , Johnson JC , Boehm K , Rice D , Kondrashova T . Impact of the clinical ultrasound elective course on retention of anatomical knowledge by second‐year medical students in preparation for board exams. Clin Anat. 2015;28(2):156–163. 10.1002/ca.22494 25534185

[14] Chen WT , Kang YN , Wang TC , Lin C‐W , Cheng C‐Y , Suk F‐M , et al. Does ultrasound education improve anatomy learning? Effects of the parallel ultrasound hands‐on (PUSH) undergraduate medicine course. BMC Med Educ. 2022;22(1):207. 10.1186/s12909-022-03255-4 PMC8962240 35346161

[15] Knobe M , Carow JB , Ruesseler M , Leu BM , Simon M , Beckers SK , et al. Arthroscopy or ultrasound in undergraduate anatomy education: a randomized cross‐over controlled trial. BMC Med Educ. 2012;12(1):85. 10.1186/1472-6920-12-85 22958784 PMC3473305

[16] Griksaitis MJ , Sawdon MA , Finn GM . Ultrasound and cadaveric prosections as methods for teaching cardiac anatomy: a comparative study. Anat Sci Educ. 2012;5(1):20–26. 10.1002/ase.259 22069248

[17] Hammoudi N , Arangalage D , Boubrit L , Renaud MC , Isnard R , Collet JP , et al. Ultrasound‐based teaching of cardiac anatomy and physiology to undergraduate medical students. Arch Cardiovasc Dis. 2013;106(10):487–491. 10.1016/J.ACVD.2013.06.002 23911833

[18] Bell FE , Neuffer FH , Haddad TR , Epps JC , Kozik ME , Warren BC . Active learning of the floor of mouth anatomy with ultrasound. Anat Sci Educ. 2019;12(3):310–316. 10.1002/ASE.1839 30414266

[19] Stringer MD , Duncan LJ , Samalia L . Using real‐time ultrasound to teach living anatomy: an alternative model for large classes. N Z Med J. 2012;125(1361):37–45. Available from: https://europepmc.org/article/MED/22960714. Accessed 27 Feb 2025. 22960714

[20] Grignon B , Oldrini G , Walter F . Teaching medical anatomy: what is the role of imaging today? Surg Radiol Anat. 2016;38(2):253–260. 10.1007/s00276-015-1548-y 26298830

[21] Estai M , Bunt S . Best teaching practices in anatomy education: a critical review. Ann Anat. 2016;208:151–157. 10.1016/j.aanat.2016.02.010 26996541

[22] Govender S , Cronjé JY , Keough N , Oberholster AJ , van Schoor AN , de Jager EJ , et al. Emerging imaging techniques in anatomy: for teaching, research and clinical practice. In: Shapiro L , Rea PM , editors. Biomedical visualisation. Advances in experimental medicine and biology. Volume 1392. Cham: Springer; 2023. p. 19–42. 10.1007/978-3-031-13021-2_2 36460844

[23] Smith CF , Barfoot S . Implementation of ultrasound in anatomy education. In: Rea PM , editor. Biomedical visualisation. Advances in experimental medicine and biology. Volume 1317. Cham: Springer; 2021. p. 111–130. 10.1007/978-3-030-61125-5_6 33945134

[24] Ma IWY , Steinmetz P , Weerdenburg K , Ma IWY , Woo MY , Olszynski P , et al. The Canadian medical student ultrasound curriculum: a statement from the Canadian ultrasound consensus for undergraduate medical education group. J Ultrasound Med. 2020;39(7):1279–1287. 10.1002/jum.15218 31943311 PMC7317450

[25] Schulz KF , Altman DG , Moher D . CONSORT 2010 statement: updated guidelines for reporting parallel group randomised trials. BMJ. 2010;340(7748):c332. 10.1136/BMJ.C332 20332509 PMC2844940

[26] Apenteng PN , Lilford R . UK medical education should include training in point‐of‐care ultrasound. BMJ. 2023;380:574–575. 10.1136/bmj.p574 36898723

[27] Patten D . Using ultrasound to teach anatomy in the undergraduate medical curriculum: an evaluation of the experiences of tutors and medical students. Ultrasound. 2015;23(1):18. 10.1177/1742271X14542173 27433233 PMC4760561

[28] Teichgräber UKM , Meyer JMA , Nautrup CP , Von Rautenfeld DB . Ultrasound anatomy: a practical teaching system in human gross anatomy. Med Educ. 1996;30(4):296–298. 10.1111/J.1365-2923.1996.TB00832.X 8949542

[29] Ratti S , Haji‐Hassan M , Călinici T , Drugan T , Bolboacă SD . Effectiveness of ultrasound cardiovascular images in teaching anatomy: a pilot study of an eight‐hour training exposure. Int J Environ Res Public Health. 2022;19:3033. 10.3390/ijerph19053033 35270725 PMC8910278

[30] Jurjus RA , Dimorier K , Brown K , Slaby F , Shokoohi H , Boniface K , et al. Can anatomists teach living anatomy using ultrasound as a teaching tool? Anat Sci Educ. 2014;7(5):340–349. 10.1002/ASE.1417 24327576

[31] Hussey D , Shaw AV , Brian PL , Lazarus MD . Learning head and neck anatomy through a radiological imaging platform. MedEd Portal. 2022;18:11230. 10.15766/mep_2374-8265.11230 PMC8907321 35342790

[32] Wang TC , Chen WT , Kang YN , Lin CW , Cheng CY , Suk FM , et al. Why do pre‐clinical medical students learn ultrasound? Exploring learning motivation through ERG theory. BMC Med Educ. 2021;21(1):438. 10.1186/s12909-021-02869-4 34412610 PMC8375120

