# Supplementary figures and images for: A randomized controlled study on medical students learning anatomy through hands‐on ultrasound
Source: Anat Sci Educ. 2025 Jul 2;18(9):948–60. doi: 10.1002/ase.70078 (PMC12413477; doi:10.1002/ase.70078)

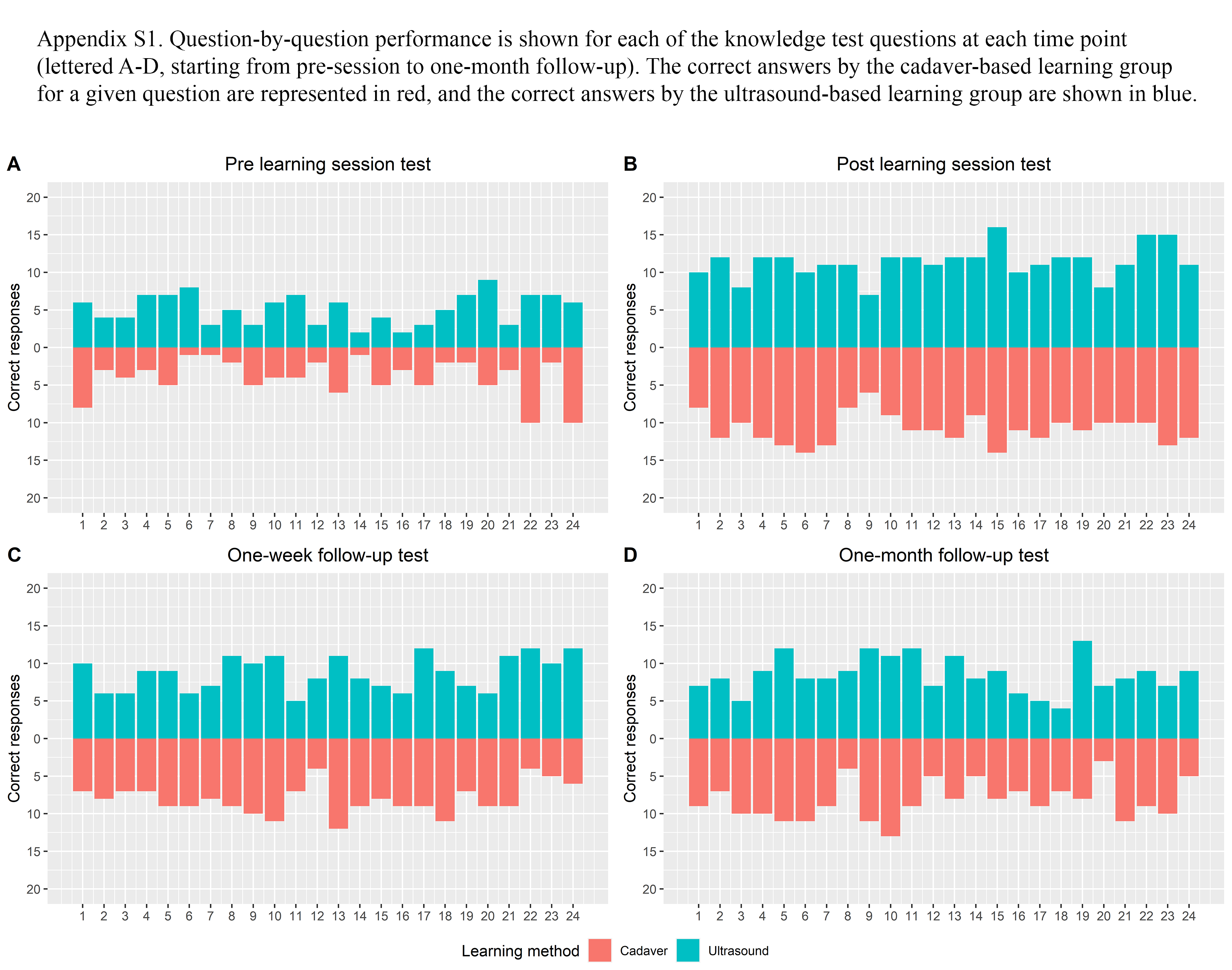

Supplement: Supplementary file 1 — Appendix S1. [file ASE-18-948-s001.png]
