# Supplementary material for: A randomized controlled study on medical students learning anatomy through hands‐on ultrasound
Source: Anat Sci Educ. 2025 Jul 2;18(9):948–60. doi: 10.1002/ase.70078 (PMC12413477; doi:10.1002/ase.70078)
Supplement: Supplementary file 2 — Appendix S2. [file ASE-18-948-s002.docx]

| Appendix S2. Mann-Whitney U Test scores for the comparison of Likert questionnaire answers between the cadaveric and ultrasound groups. | | |
| --- | --- | --- |
| **Question** | **p-value** | **U-statistic** |
| **Head and Neck Module** |  |  |
| This was an effective format of learning anatomy of this region | 0.468 | -34 |
| This was an enjoyable format of learning anatomy of this region | 0.467 | -33.5 |
| I felt actively involved in the learning process during this module | 0.145 | -57.5 |
| The learning modality was clinically relevant for this region | 0.346 | -41.5 |
| The quiz questions were reflective of the content demonstrated in this module | 0.774 | -20 |
| I feel confident in my anatomy knowledge regarding this region | 0.251 | -50 |
| **Upper Limb Module** |  |  |
| This was an effective format of learning anatomy of this region | 0.593 | 8.5 |
| This was an enjoyable format of learning anatomy of this region | 0.692 | 3 |
| I felt actively involved in the learning process during this module | 0.370 | -38.5 |
| The learning modality was clinically relevant for this region | 1.000 | -10 |
| The quiz questions were reflective of the content demonstrated in this module | 0.630 | -26 |
| I feel confident in my anatomy knowledge regarding this region | 0.439 | -37.5 |
| **Abdomen Module** |  |  |
| This was an effective format of learning anatomy of this region* | 0.036 | 56.5 |
| This was an enjoyable format of learning anatomy of this region | 0.794 | -2.5 |
| I felt actively involved in the learning process during this module | 0.358 | -39 |
| The learning modality was clinically relevant for this region | 0.599 | -27 |
| The quiz questions were reflective of the content demonstrated in this module | 0.431 | 16.5 |
| I feel confident in my anatomy knowledge regarding this region | 0.865 | -16.5 |
| **Regarding the entire learning session** |  |  |
| I had a satisfactory learning experience | 0.860 | -4 |
| I feel more confident in operating a handheld ultrasound device | <0.001 | -183.5 |
| I am interested in additional ultrasound learning opportunities | 0.219 | -48.5 |
| I think hands-on ultrasound should be part of my anatomy education | 0.149 | -59 |
